# Drug use and antiretroviral therapy (ART) interactions: a qualitative study to explore the knowledge, beliefs, adherence, and quality of life of people living with HIV taking ART and illicit drugs

**DOI:** 10.1186/s12981-020-00279-y

**Published:** 2020-05-24

**Authors:** María José Fuster-RuizdeApodaca, Vanessa Castro-Granell, Ana Laguía, Ángeles Jaén, Santiago Cenoz, María José Galindo

**Affiliations:** 1 Spanish Interdisciplinary Aids Society (Sociedad Española Interdisciplinaria del Sida, SEISIDA), Madrid, Spain; 2 Fac. de CC. de la Salud, Departamento de Psicología, Universidad Nacional de Educación a Distancia (UNED), Madrid, Spain; 3 Doctoral Program in Pharmacy, Granada University, Granada, Spain; 4 Hospital Marina Baixa, Villajoyosa, Alicante, Spain; 5 Universidad Rey Juan Carlos (URJC), Facultad de Psicología, Madrid, Spain; 6 Fundació de Docència i Recerca Mutua Terrassa, Terrassa, Barcelona, Spain; 7 ViiV Healthcare, Tres Cantos, Madrid, Spain; 8 Hospital Clínico Universitario de Valencia, Valencia, Spain; 9 Hospital Marina Baixa, Av. Alcalde En Jaume Botella Mayor, 7 Villajoyosa, 03570 Alicante, Spain

**Keywords:** HIV, Illicit drugs, Antiviral therapy, Drug–drug interactions, Treatment adherence, Quality of life

## Abstract

**Background:**

To explore the use of illicit drugs by people living with HIV (PLHIV) taking antiretroviral therapy (ART) and their relationship with variables relevant to the management of HIV infection, such as knowledge and beliefs about drug–drug interactions (DDIs), ART adherence, quality of life (QoL), and use of health-care resources.

**Methods:**

21 PLHIV in Spain who concomitantly took illicit drugs and ART participated in this qualitative study. Eight experts collaborated in the design of the semi-structured interview guide which explored the following topics: illicit drug use, knowledge and beliefs about DDIs and their impact on ART adherence, the effects of using illicit drugs on health, QoL, and use of health-care resources. Four of those experts, who were PLHIV and members of the executive boards of non-government organizations (NGOs) from four Spanish regions, recruited the participants through their NGOs and carried out the face-to-face interviews. Content analysis of the qualitative data was conducted with the support of the MAXQDA 12 program.

**Results:**

Participants were mainly men (85.7%) and only 14.3% of them were heterosexual. Content analysis showed that the most frequently consumed illicit drugs were poppers, cocaine, and cannabis. Participants were polydrug users and this was, in many cases, prior to HIV diagnosis. Most participants presented theoretical potential moderate DDIs that would require monitoring. More than three quarters of them were not aware of these DDIs. Participants reported interactive toxicity beliefs that lead to intentional nonadherence behaviors. In most cases (n = 17), the participant’s doctor knew about their drug use, however only six of them had had an open dialogue with their physician about it. Illicit drug use led to some health-related problems, mainly sexually transmitted infections. A positive QoL’s self-perception was found among several participants that used recreational illicit drugs.

**Conclusions:**

Adequate information about DDIs and clues about how to manage ART when PLHIV are using illicit drugs could reduce the negative effects of such interactions and improve ART adherence and QoL.

## Background

The use of illicit drugs in a mainly “recreational” context has increased worldwide during the last two decades, this constitutes a global public health problem. [1] This is most prevalent in the subgroup of the population of men who have sex with men (MSM), with significantly higher figures than those observed in the general population. [2, 3] Studies have revealed “polydrug use” (use of more than one illicit drug at the same time or over a period of time), a particularly predominant phenomenon in MSM with HIV [4, 5].

Illicit drug use has been associated with a higher prevalence of unprotected sexual behavior and, therefore, with a greater acquisition of HIV and other sexually transmitted infections (STIs) [3, 6]. The use of illicit drugs may also have different implications in diverse health-related variables of PLHIV. One of them is the emergence of potential interactions between the anti-retroviral drugs and other drugs [7], which could lead to failure of the therapeutic anti-retroviral treatment (ART) or, indeed, to toxicity. Particularly, polydrug use could be associated with severe clinical consequences [8].

Some studies have also found an association between the consumption of illicit drugs and nonadherence to ART in HIV patients, thereby limiting the benefits of treatment [9, 10]. The most studied effects are the lack of adherence caused by intoxication or secondary forgetfulness after taking illicit drugs (unintentional nonadherence) [11]. However, apart from unintentional nonadherence, people can intentionally discontinue medication when they consume alcohol or drugs because they believe that taking both would be harmful (beliefs in toxicity by interaction), perceiving a dilemma of choosing between the continuation of ART or stop consuming illicit drugs [11]. The few investigations studying intentional medication nonadherence showed that it predicted poorer ART adherence and poorer treatment outcomes (e.g., unsuppressed viral load) [11].

In addition to the emergence of interactions and the impact on ART-adherence, illicit use in PLHIV can lead to other clinical consequences such as a worse link to health care and a worse prognosis of the progression of the infection [12, 13]. Some authors also claim that the health economy may deteriorate, with increased hospitalizations and visits to outpatient centers and emergency units, increases in doses or changes in prescriptions, or increases in or more invasive diagnostic testing [14].

Illicit drug use can also have a negative impact on the quality of life (QoL) of PLHIV. The results of a review of studies on the determinants of the QoL of PLHIV showed that illicit drug use was associated with poorer physical and mental health [15]. However, most studies have focused on people who inject or had injected drugs (mainly heroin). The few existing studies on recreational illicit drugs have shown that mental health is affected more than physical health as a result of illicit drug use, and that amphetamines and methamphetamines are associated with greater deterioration [16].

Based on the above background, this research has the following objectives: (a) to explore illicit drug use and its context in PLHIV in Spain who took diverse types of illicit drugs and who were on ART, (b) to explore these people’s knowledge and beliefs about the possible interactions between illicit drugs and ART (drug–drug interactions, DDIs) as well as their implications in adherence to ART, and (c) to examine the QoL of PLHIV who use illicit drugs as well as the use of health-care resources. To achieve these goals, a qualitative study was conducted. This design allowed us to conjointly explore relevant variables for managing the infection that needs further study, having direct information from the target population’s opinion.

## Methods

### Design and sampling

The present qualitative study was the first phase of a broader sequential exploratory research conducted in Spain between 2016 and 2018. We interviewed 21 PLHIV who took a diverse type of illicit drugs and had taken ART for at least 1 year. We decided on the profile of PLHIV to be interviewed through theoretical sampling guided by the results of the focus group described in the procedure section. Experts participating in the focus group agreed on the need to interview the following demographic and epidemiological profiles of PLHIV: men, women, different ages, MSM (including sex workers), heterosexual, transgender women, and migrants. Moreover, they agreed with the relevance of including PLHIV who used diverse types of drugs to allow us comparisons in the variables under study. According to previous results in Spain [6], the following patterns of use of illicit drugs were established: (i) Recreational drugs and sexualized drugs. Recreational drugs are those used mostly in night parties such as cocaine, ecstasy, *speed*, crystal, mephedrone, methylenedioxymethamphetamine (MDMA), gamma-hydroxybutyric/gamma-butyrolactone (GHB/GBL), ketamine, among others; while sexualized drugs are those used exclusively in a sexual context such as nitrites (*poppers)*; (ii) traditional illicit drugs, which are those linked to social exclusion such as heroin or crack cocaine.

Afterwards, we conducted an observational cross-sectional study in which 1.401 PLHIV were recruited from 12 Spanish regions [17]. Lastly, we conducted a multicenter observational retrospective cohort research in which two cohorts of PLHIV who used and who did not use recreational drugs were recruited (N = 275 PLHIV) from four hospitals from three Spanish regions. These studies delved into the research questions and hypotheses posed using different methodological approaches.

This research was conducted using a paradigm of community-based participatory research, so that members of the population under study were directly implicated in all its phases [18, 19]. Thereby, PLHIV were involved in different stages of the study. One of the principal researchers was a person with HIV. Moreover, an advisory group made up of eight experts from diverse disciplines and settings, including community-based organizations linked to the population under study, guided the research, and participated in its different phases. Besides, recruitment and face-to-face interviews were performed by the experts living with HIV members of the advisory group. The research team discussed all findings of the research with the expert community-based advisory board.

The Ethics Committee of the Hospital Clínico of Valencia approved the research protocol. The participants were informed of the objectives of the study and written informed consent was obtained. None of the selected participants refused to participate.

### Procedure

Firstly, we conducted a focus group with experts who were working in health-care and in the community. This group discussed the state of the problem under study and delimited the relevant issues to be explored. This focus group lasted approximately 3 h.

Once the group discussed the theoretical contents, we assigned some time to debate with the experts methodological and procedural issues regarding the next steps of the study. Firstly, we designed the specific composition of the sample according to the criteria that they had previously agreed. The group accorded that the four experts that were PLHIV and members of the executive boards of non-government organizations (NGOs) from four Spanish regions (Barcelona, Madrid, Seville, and Valencia) recruited the participants through their NGOs and carried out the face-to-face interviews. This decision was taken based on the fact that their proximity with the interviewees could encourage them to speak confidently about the sensitive issues explored in the interview. Afterwards, we distributed the theoretical profiles of PLHIV among these four experts according to the main profile of PLHIV they attended in their NGOs. Then, the four experts selected potential participants among their NGOs’ users. These four experts were individually trained by a researcher of the team expert in qualitative methodology. The collaborating experts explained the goals of the study to the pre-selected participants, requesting their participation, and obtaining their signed informed consent. Interviews were conducted privately in the NGOs between April and June 2016 and lasted approximately 1 h each. Continued supervision and advice were provided to these four experts while the interviews were conducted.

### Instrument

**Fig. 1:**
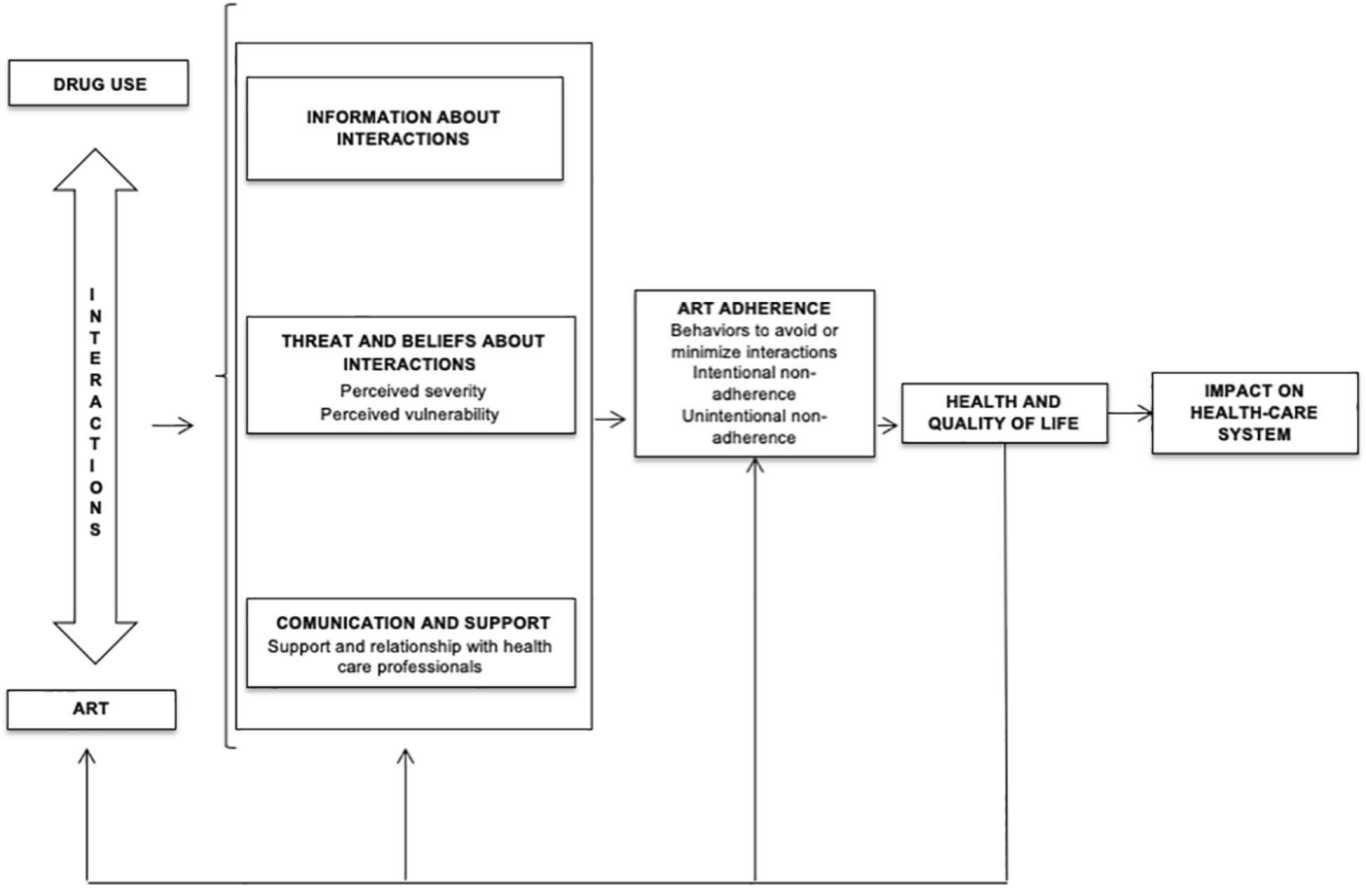
Conceptual framework showing the domains analyzed in the study

We designed the structure of the script of the interview based on the conceptual framework of the study (Fig. 1). Besides, it was based on the literature about the determinants of health behaviors in HIV [20, 21], on the relevant domains identified by the experts in the focus group, and on the research questions of the study about such domains. The interview script contained the following five sections: (i) Health data and the use of medications. It included questions related to ART and other prescribed medications the participants had; (ii) Drug use. It explored the illicit drugs and other substances that the participants took, the reasons to consume them, the frequency and patterns of consumption, and the relationship between illicit drug use and HIV diagnosis; (iii) Knowledge and beliefs about interactions. It included questions aimed at finding out the interviewee’s degree of knowledge about DDIs, beliefs about it, and communication with healthcare providers about their use of illicit drugs; (iv) Behaviors adopted to alleviate DDIs and the impact on adherence. It explored adherence behaviors and the kind relationship between it and the use of illicit drug use; (v) The impact of drug use on health, QoL and the health system. It contained questions aimed to explore how was the interviewee’s perception of health and QoL, whether they had experienced negative health consequences or had to increase their use of healthcare resources due to the use of illicit drugs. The specific questions asked in the interview can be found in a supplementary file (Additional file 1). The script was reviewed and agreed on by the group of experts and members of the research team. Three initial interviews were conducted in order to pilot the interview. Besides, before the interview, we collected demographic data and limited health data in written form.

### Data analysis

We performed content analysis of the interviews [22] using MAXQDA 12 software. For this purpose, the interviews were verbatim transcribed, reviewed for accuracy, and coded. A book of deductive codes was developed, following the map of the design of the study and the research questions [23]. This process allowed us to rate the information saturation in the main topics of the interview [24]. Next, the codes were grouped into categories, which were formed mainly using a theory-driven approach performed by the main analyst. A second researcher then coded 10 interviews (nearly 50%). The mean percentage of agreement of the presence and frequency of categories in each segment was 69.75% (± 7.6) and Cohen’s mean kappa index of agreement was 0.68 (± 0.9). Any inconsistencies among judgements in categorization were resolved by consensus. All the research team reviewed and approved the final categorizations.

To analyze patterns and differences in the categories according to the participant’s profile of use of illicit drugs we used the mixed methods tool of the MAXQDA 12 software. This profile was established according to the findings of Folch et al. [6].

To determine the potential theoretical interaction between the ART and the illicit drugs the participants used, we checked the daily-practice interaction database http://www.hiv-druginteractions.org by the University of Liverpool. We analyzed clinically significant DDIs (moderate and contraindicated), that is, those requiring close monitoring, modification of dosage or adaptation of the therapeutic range of the drugs involved, and those whose combination would be contraindicated due to their potential to cause serious adverse effects. We used the mixed-methods tools of the MAXQDA 12 software to compare the discourses in the knowledge, beliefs, and adherence behaviors according to the DDIs’s participant’s classification.

## Results

### Characteristics of the participants

Table 1 shows the characteristics of the participants. As can be seen, most were MSM with a mean age around 40 years old. Five participants, including three women, were selected for having a profile of consumption of traditional illicit drugs (mainly heroin), whereas the profile of the rest was of recreational and/or sexualized illicit drug use.

**Table 1:** Characteristics of the participants

	N (%)
N	21
Gender	
Males	18 (85.7)
Females	3 (14.3)
Country of birth	
Spain	19 (90.5)
Other European country	1 (4.7)
Other countries	1 (4.7)
Sexual behavior	
Heterosexual	3 (14.3)
Homosexual	16 (76.2)
Bisexual	2 (9.5)
Educational level	
Primary education	2 (9.5)
Secondary education	9 (42.9)
University degree	10 (47.6)
Other	0 (0)
Work situation	
Working (with a legal contract)	11 (52.4)
Unemployed	6 (28.6)
Retired/work impairment	4 (19)
Others	0 (0)
Monthly income	
< 1000€	12 (57.1)
1000–1499€	6 (28.6)
1500–1999€	1 (4.8)
> 2000€	1 (4.8)
Missing datum	1 (4.8)
Transmission route	
Unprotected sexual intercourse	16 (76.2)
Sharing injecting materials	4 (19)
Unknown	0 (0)
Other	1 (4.8)
Age in years (Mean ± SD)	40 ± 11.1
Duration of infection in years (Mean ± SD)	14.2 ± 8.9
Years taking ART (Mean ± SD)	10.2 ± 8.5

*ART* antiretroviral therapy

### Illicit drug use

**Fig. 2:**
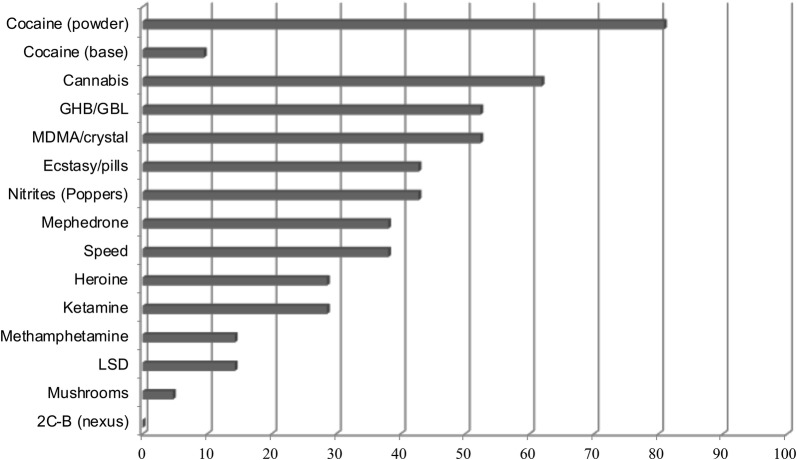
Illicit drugs consumed by the participants

The illicit drugs most consumed by the participants were poppers, cocaine, cannabis, MDMA, and GHB/GBL (Fig. 2). The average number of different illicit drugs that were consumed was 5.33 (± 1.95). Participants commonly reported consuming several illicit drugs during the same outing or party. Most of the people interviewed had consumed illicit drugs before the diagnosis of HIV (n = 17).

In addition to drugs, participants consumed some medicines without a prescription; the most frequently used drugs being erection enhancers (n = 11). Anabolic steroids (n = 2) and supplements (n = 4) were also consumed, but with less frequency.

It was observed that the frequency of consumption was very heterogeneous and varied depending on the type of illicit drug consumed. Cannabis was the illicit drug that the participants who consumed it reported to be consumed daily. Participants who use traditional illicit drugs (heroin or cocaine base) reported using such illicit drugs several times per week. However, those who used recreational illicit drugs reported lower frequencies of consumption (once per week or a few times per month but not weekly).

Regarding the modes of consumption, the most common were inhalation (n = 15) and oral (n = 9). Six participants, half of them with a profile of traditional illicit drug use and half with a recreational illicit drug use profile, reported having injected drugs. Besides heroin and cocaine, the illicit drugs consumed by injection were mainly mephedrone and methamphetamine. Some participants (n = 3) reported using rectal administration for certain illicit drugs such as mephedrone, cocaine, or methamphetamine.

### Drug-drug interactions: knowledge, beliefs, and taking ART and illicit drugs

Results showed that most participants (n = 16) presented theoretical potential moderate interactions that would require some intervention such as close monitoring, dosage adjustment or timing of administration by medical personnel. No patient presented contraindicated DDIs. The mean DDIs per participant was 2.95 (± 3.18).

Most participants with theoretical potential moderate DDIs (n = 16) were unaware of having such DDIs. We explored the participants’ beliefs about the DDIs. We found a predominant discourse about the perception of negative consequences, especially in the beliefs that mixing illicit drugs and ART can create toxicity (interactive toxicity beliefs) and that this may decrease the effectiveness of ART. However, and despite these predominant beliefs, there was no perception of personal risk or threat or else participants minimized or relativized the possible negative consequences. The main sources of the lack of perceived risk were ignorance and the fact of not having experienced negative health consequences. The degree of knowledge and beliefs did not differ among the participants’ profiles of illicit drug use in this study. However, the absence of perception of personal risk was more present in those who had a profile of use of traditional illicit drugs.

**Fig. 3:**
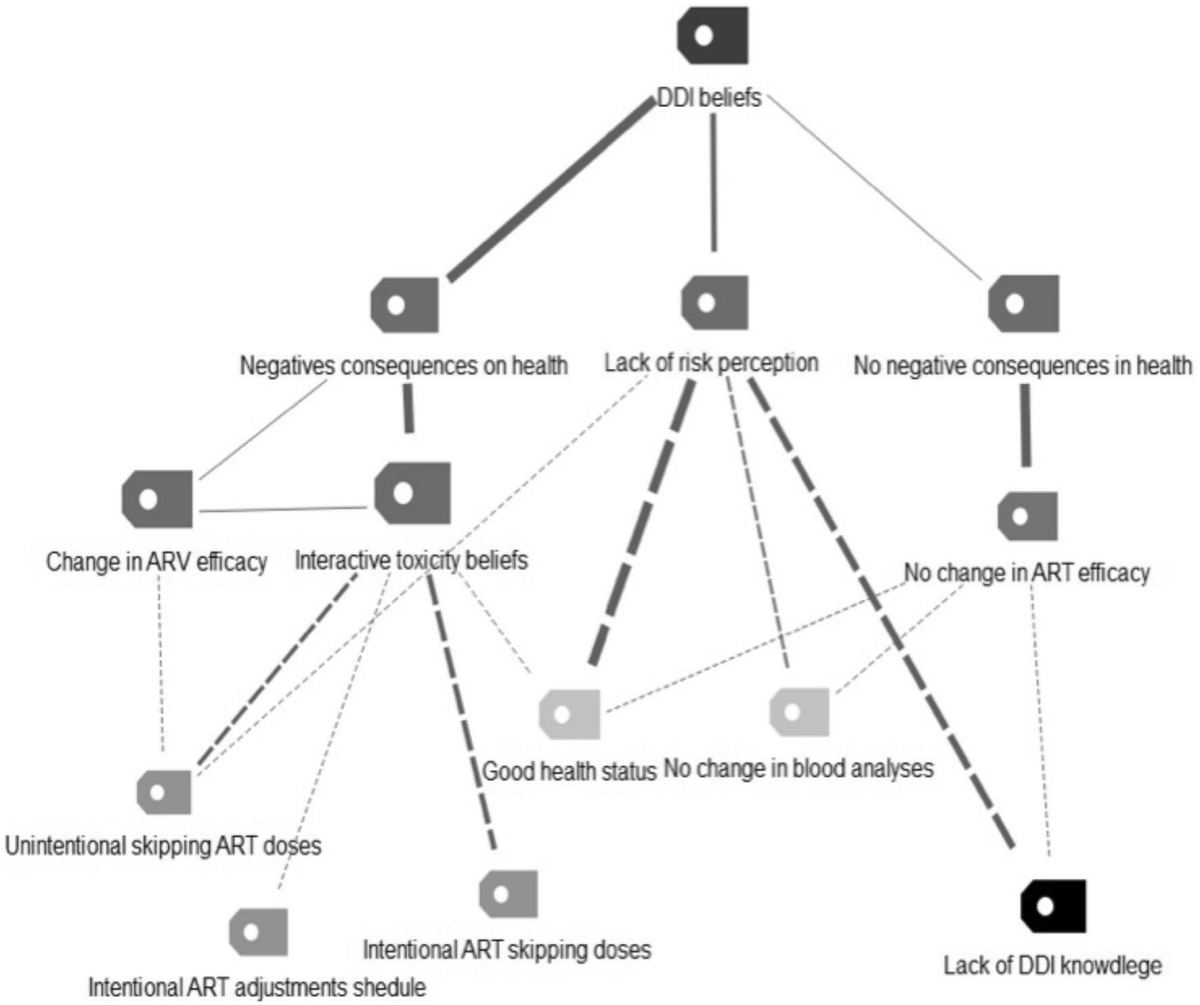
Map of the relationships between categories related to DDIs beliefs

Illicit drug use was associated with unintentional nonadherence, as more than half of the participants omitted doses or delayed their medication schedules unintentionally because they were under the influence of illicit drugs. However, some participants reported omitting ART doses or intentionally adjusting their schedules to prevent possible negative effects of taking ART and illicit drugs together. These categories of discourse were related to toxicity beliefs. Table 2 shows the categories and fragments of interviews that illustrate them. The map of relationships between categories is shown in Fig. 3.

**Table 2:** Knowledge and beliefs about interactions and impact on adherence

Categories	N	Segment
Ignorance of ART-drug interactions	14	No, I had not considered the issue of drugs, I mean, I bear in mind the fact that drugs are not good(!), otherwise I do not think that they would be prohibited. I mean the damage they can do at the level… at the level of memory, and the level of intelligence, and all that. But the truth is that I never considered the subject of interactions… (Interview V-3)
Beliefs about ART-drug interactions^a^		
Negative consequences		
Interactive toxicity beliefs	11	It’s like I said earlier, it is chemistry. That is, chemistry with chemistry, well bad things can happen… (Interview S-4). What could happen? It’s like the movie… the first film of Batman, the Joker’s combination of products, if you get the bad combination, you can die. Also, it depends on each body (Interview B-2)
Decreased effectiveness of ART	9	Well, I imagine that defenses would decrease, anyway… ART would somehow eventually stop being effective (Interview S-5).
Absence of negative consequences	4	I think there is no problem when you take drugs with medication (Interview V-5)
Perception of threat or vulnerability		
Perception of threat to health	11	Well,. sometimes I say: you gotta die of something! You know? (laughs). And sometimes I say we must live! And sometimes I say… I don’t know… Well! In the long term, it must leave its mark, no?… (Interview M-1)
Absence of perception of health risk	8	Nothing… I don’t notice anything at all. The effects of the drugs are exactly the same as those I had before having HIV and before taking treatment… Because in the months that I have been… in the years I’ve been in treatment, I have taken drugs at the same time, and my levels all have continued to be OK (Interview S-2)
Minimization or relativization of the risk	2	…Will it have interactions? The same as if you take three coffees and then you do not sleep at night, you know? I mean: it has interactions, well, yes, for the same reason, because it’s chemistry with chemistry and that cannot be good. But very, very, serious interactions, maybe you have to be very, very addicted to drugs… I am not very, very addicted, although I consume many types… (Interview S-4)

*ART* antiretroviral therapy

^a^Responses that allowed multiple coding

### Communication with health care professionals on the use of illicit drugs

Most of the participants (n = 17) claimed that their HIV specialist knew about their use of illicit drugs. However, only six of these participants reported having had an open talk about it. The analysis of the categories’ relationships showed that open talk about using illicit drugs was related to interactive toxicity beliefs about DDIs.

### Illicit drug use, health and quality of life

More than half of the participants (n = 14) reported some physical health problems as a consequence of their use of illicit drugs. The most frequent (n = 8) were STIs because they had unprotected sexual relationships while they were using drugs. Other health problems reported less frequently were overdoses, hepatitis, dental problems, fatigue, and sexual dysfunction.

Some people also indicated that illicit drug use had negative effects on the social sphere, such as relationships or work, and/or the psychological sphere, such as an emotional decline after consuming illicit drugs or anxiety disorders. Whereas the participants who related problems in the social sphere were mostly those that used traditional illicit drugs, participants that used mainly recreational illicit drugs reported problems in the psychological sphere.

Most participants who used recreational illicit drugs expressed a positive perception of their QoL (n = 16). In case of participants who used traditional illicit drugs the discourse was more negative, and they offered different reasons for dissatisfaction with life. Table 3 shows the categories found and fragments of speech illustrating them.

**Table 3:** Impact of drugs and perceived quality of life

Categories	N	Segment
Impact of drugs^a^		
Negative impact on physical health	14	Of course, I’ve caught everything, everything!…that is, every 3 months I had to go to Center for Sexually Transmitted Diseases like… My penis festers, I don’t know what festers… my throat… I have…! (Interview S-4)
Negative impact on the social sphere	11	Then the couple relationships, the second-to-last… the second-to-last misfired a lot due to drug use because, at that time, I was working at night and I consumed continuously on Thursday, Saturday, Sunday and then during the week, you’re wrecked! Besides, sexually it does not work. Your partner more or less has to pay for your bad temper… (Interview M-3)
Negative impact on the psychological sphere	6	Well, for starters I realized, when I started taking drugs, that they leave you… that is, sometimes it isn’t worthwhile, I mean, that even if you keep doing it, I am aware that the next day, I am psychologically in a very bad way. Psychologically they leave you wrecked… that is, when I take drugs, the next day, I cannot be alone, I cannot be alone! I have to be with someone, because it reminds me a lot, a lot of the first year when I was infected. (Interview V-2)
Quality of life		
Positive discourse	8	The day after I take drugs I feel terrible, but I want to experiment… But my quality of life very well. Socially great. With the family super good, because I have everything super normalized… (Interview V-2)
Negative discourse	8	Because of the abstinence syndrome, I have a horrible time, very bad. Several days a week I feel terrible. Therefore my quality of life is awful (Interview B-1)
Ambivalent discourse: general positive rating but with specific domains of dissatisfaction	5	… I’m much better at a personal level with myself. And I’m OK, if I didn’t have this (HIV), about which people must be informed, I would be fine. Health-wise, I feel good, regarding future prospects, I am well. The only thing, I lack a little bit of environment, which must still be built (Interview B-4)

*ART* antiretroviral therapy

^a^Responses that allowed multiple coding

### Impact of illicit drug use on the health-care system

In some participants (n = 11), the problems arising from illicit drug use had led to having to visit different health services (such as the emergency unit), increased frequency of visits to the doctor, and the need for hospitalization. The increase in the use of health-care resources was more frequent among persons that used mainly traditional illicit drugs (n = 5), although there were some participants that used recreational illicit drugs who had had to visit the emergency unit or who had increased the frequency of their medical visits (n = 3) as a result of their illicit drug use.

## Discussion

This research studied illicit drug use by PLHIV who take ART and its relationship with relevant variables for managing the infection, such as knowledge and beliefs about DDIs, the implications in adherence to ART, and the effects of illicit drug use on health, QoL, and the use of health-care resources.

Firstly, the results have shown that the participants were illicit drug polyconsumers, mainly of recreational and/or sexualized illicit drugs. The most frequently consumed illicit drugs were poppers, cannabis and cocaine. These results are similar to those of other studies carried out both in Spain and other countries, mainly in MSM but also in PLHIV [2–4, 10].

Secondly, the results have shown a low level of knowledge about DDIs, even among PLHIV with theoretical potential moderate DDIs that would be acceptable if they were monitored. Ignorance as well as good health status was found to be associated in the discourses with the lack of perceived risk of such DDIs. The literature points out that these variables are relevant determinants of health behavior [20, 21]. The predominant beliefs about DDIs were that these interactions can produce toxicity or decrease the effectiveness of ART. Toxicity beliefs were associated with more behaviors of intentional nonadherence. This relationship had already become clear in other studies of PLHIV who consumed alcohol [25] and drugs [11]. Furthermore, it was found that an open dialogue with the HIV specialist was also associated with interactive toxicity beliefs. The potential association of these beliefs with intentional nonadherence reveals the importance of communication and medical advice to help PLHIV to make appropriate use of the ART when they consume drugs. However, to our knowledge, this issue has not yet been studied in PLHIV.

The results also showed that illicit drug use had negative effects on some health variables, mainly on the acquisition of STIs. This finding is in line with the scientific evidence that shows that illicit drug use is associated with an increase in the number of casual sexual partners and a greater likelihood of not using condoms with them [6, 10, 26]. In addition, and in line with the indications of other studies [14], we also found that health problems arising from illicit drug use sometimes led to increased visits to various medical services.

The participants in this study reported different psychological and social problems, although the latter were more frequent in users of traditional illicit drugs. In users of recreational illicit drugs, we found a positive discourse on QoL. These results could be related to the reasons pointed out by some authors for consuming illicit drugs in MSM with HIV, such as improving their self-esteem, emotional needs and feelings of loneliness, and social and sexual disinhibition, among others [27–29]. However, it has also been shown that users of recreational illicit drugs are currently well adapted to their socio-familial and work environment, which is perceived as a lack of negative consequences derived from consumption [30]. Future studies should examine possible differences in the QoL of PLHIV based on their demographic, health, and illicit drug use profile.

The results of our study should be interpreted in the light of its limitations; mainly, those due to the design and sample size which prevents generalizability of results. However, thanks to the design of the study, we had access to direct information of the target population’s opinion and to the advice of experts, many of whom belonging to the community. In addition, we performed various types of triangulation, which provide evidence of validity [31]. On the one hand, we carried out triangulation of the data, using different sources and examining the topic in different local environments and typologies of PLHIV. On the other hand, we performed researcher triangulation as different people interviewed the participants, with the advantage of the fact that they belonged to the community.

## Conclusions

PLHIV taking ART and illicit drugs reported interactive toxicity beliefs that have an impact on intentional nonadherence. Providing patients with adequate information about DDIs and clues about how to manage ART when they also use drugs could improve ART adherence and QoL. Such information should correct possible misinformation about interactive toxicity beliefs. Illicit drug use seems to have negative effects on other health- and psychosocial-related variables. Future studies should examine in depth the impact of recreational illicit drug use on QoL, and the kind of interventions needed to improve it in PLHIV that show problematic illicit drug use and to prevent its deterioration in those with non-problematic illicit drug use.

## Supplementary information

**Additional file 1: Table S1.** Script of semi-structured interview.

## Data Availability

All relevant data are within the manuscript. The datasets collected and/or analyzed during the current study are available on request from the author.

## Abbreviations

ART: Antiretroviral therapy
DDIs: Drug–drug interactions
GHB/GBL: Gamma-hydroxybutyric/gamma-butyrolactone
HIV: Human immunodeficiency virus
MDMA: Methylenedioxymethamphetamine
MSM: Men who have sex with men
PLHIV: People living with HIV
NGOs: Non-governmental organizations
QoL: Quality of life
STIs: Sexually transmitted infections
