# Supplementary material for: Drug use and antiretroviral therapy (ART) interactions: a qualitative study to explore the knowledge, beliefs, adherence, and quality of life of people living with HIV taking ART and illicit drugs
Source: AIDS Res Ther. 2020 May 24;17:24. doi: 10.1186/s12981-020-00279-y (PMC7245822; doi:10.1186/s12981-020-00279-y)
Supplement: Supplementary file 1 — Additional file 1: Table S1. Script of semi-structured interview. [file 12981_2020_279_MOESM1_ESM.docx]

| **A. Health data and use of medications**   1. Which drugs are you taking for HIV? Can you tell me what dosage schedule you follow? How was this schedule defined? 2. Do you take any medication in addition to antiretroviral medication? *If so:* what prescription medications do you take? What do you take the medicines you've mentioned for? How often do you take them? 3. Who prescribed this medication you mentioned?   **B. Drug use**   1. Could you tell me what drugs you are taking? 2. Why do you take them? 3. *If several drugs are named:* How do you consume the drugs that you have named? Do you take them all together or in different situations? How often do you use them? 4. Did you take drugs before being diagnosed with HIV? *If so:* Do you somehow relate drug consumption with the fact that you've been infected?   **C. Knowledge and beliefs about interactions**   1. Can you tell me what the concept "treatment interactions" refers to? 2. Do you know the interactions of the antiretroviral treatment with different kinds of drugs? Could you tell me what you know about these interactions? 3. Where do get the information you have about the interactions between antiretroviral therapy and drugs and other medications? 4. Have you commented with your doctor that you consume drugs? What was his/her reaction? 5. What do you think can happen if drugs and HIV medications are taken together? And conversely, what do you think can happen if the medication is not taken correctly when drugs are taken? 6. What consequences do you think the interactions of drugs and medicines for HIV can have on the health of people with HIV? 7. In your case, what consequences do you think that the possible interactions of drugs and the medications you take could have? |
| --- |

Appendix (cont.) Script of semi-structured interview

| **D. Behaviors adopted to alleviate interactions and the impact on adherence**   1. How is your adherence to antiretroviral therapy? *In case of nonadherence,* in what situations do you not take the medication? What are your reasons for not taking it correctly? 2. To what extent does the fact that you take drugs influence your adherence to treatment? *In case of not taking the medication when using drugs:*     1. Do you stop taking all medication or just some medication? What are your reasons for not taking medication when you take drugs? Do you change your ART schedule or dose when you take drugs? 3. Do you do anything to avoid or alleviate the possible interactions between the treatment and the drugs when you take drugs? *If so:* what do you do? 4. Do you take the interactions into account when choosing the drugs that you consume? *If so:* How do you take them into account? That is, which choices do you make and depending on what?   **E. Impact of drug use on health, quality of life, and the health system**   1. How is your health now? 2. Have there been any negative consequences for your health since you have taken drugs? *If so*: what are those consequences? 3. Have you ever had any sexually transmitted infection? *If so:* which ones and when? 4. How have the health problems you've had changed your regular medical follow-up? *Explore the following issues:*     1. Have you had to visit the doctor more frequently than usual as a result of these health problems? How often have you had to go?    2. Have you had to be hospitalized or visit the emergency unit as a result of these health problems? 5. In general, how would you rate your quality of life? 6. Would you like to add any comment or question that we have not addressed? |
| --- |
